# IMPLEMENTATION OF THE PAIN MANAGEMENT CLINICAL PRACTICE GUIDELINE USING THE EVIDENCE INTEGRATION TRIANGLE

**DOI:** 10.1093/geroni/igae098.1806

**Published:** 2024-12-31

**Authors:** Elizabeth Galik

**Affiliations:** University of Maryland, University of Maryland, Maryland, United States

## Abstract

To optimally implement the Pain Management CPG and change behavior among residents and staff in nursing homes we developed a theoretically based approach that incorporates the Social Ecological Model, Social Cognitive Theory and was guided by the Evidence Integration Triangle. The PAIN-CPG-EIT was implemented by a research nurse facilitator 10 hours per week for two months and then 4 hours per week for an additional four months to provide four components: Component I: Establishing and working with a stakeholder team monthly; Component II: Education; Component III: Mentoring and motivating the staff; and Component IV: Ongoing monitoring of pain assessment, diagnosis and management for residents. The first stakeholder team meeting included information about the Pain Management CPG and a brainstorming session to develop community goals. The ongoing role of the stakeholder team was to provide positive reinforcement for the staff caring for residents and support the champions. As per Component II, the research nurse facilitator provided education to staff based on the Pain Management CPG. Component III included reviewing pain related issues among residents with the staff and brainstorming about solutions, implementing treatments and adjusting care plans. In addition, contests were held to have staff share innovative approaches to addressing pain among residents. Weekly pain management tidbits were provided via email to all stakeholder members to share with staff. Component IV included the ongoing re-evaluation of pain among residents and monitoring of pain related interventions to determine if these were effective, still needed or if changes in care plans were necessary.

